# SARS-CoV-2 laboratory surveillance during the first year of the COVID-19 pandemic in southern Brazil

**DOI:** 10.1590/0037-8682-0146-2022

**Published:** 2023-01-23

**Authors:** Ludmila Fiorenzano Baethgen, Ana Beatriz Gorini da Veiga, Richard Steiner Salvato, Talita Giacomet de Carvalho, Thaiane Rispoli, Sun Hee Schiefelbein, Letícia Garay Martins, CEVS-COVID-19 Team, Zenaida Marion Alves Nunes, Anelise Praetzel Schaurich, Loeci Natalina Timm, Rosane Campanher Ramos, Cynthia Goulart Molina Bastos, Tatiana Schäffer Gregianini

**Affiliations:** 1 Laboratório Central de Saúde Pública, Centro Estadual de Vigilância em Saúde, Secretaria de Saúde do Estado do Rio Grande do Sul, Porto Alegre, RS, Brasil.; 2 Universidade Federal de Ciências da Saúde de Porto Alegre, Departamento de Ciências Básicas da Saúde - Biologia Molecular, Porto Alegre, RS, Brasil.; 3 Hospital Fêmina, Grupo Hospitalar Conceição, Rio Grande do Sul, Brasil.; 4 Projetos de Cooperação Técnica - Pan American Health Organization/World Health Organization - Secretaria Estadual de Saúde do Estado do Rio Grande do Sul, Porto Alegre, RS, Brasil.; 5 Centro de Operações de Emergência, Centro Estadual de Vigilância em Saúde, Secretaria de Saúde do Estado do Rio Grande do Sul, Porto Alegre, RS, Brasil.; 6 Centro de Desenvolvimento Científico e Tecnológico, Centro Estadual de Vigilância em Saúde, Secretaria de Saúde do Estado do Rio Grande do Sul, Porto Alegre, RS, Brasil.; 7 Centro Estadual de Vigilância em Saúde, Secretaria de Saúde do Estado do Rio Grande do Sul, Porto Alegre, RS, Brasil.

**Keywords:** Molecular diagnosis, SARS-CoV-2, SARS-CoV-2 lineages, Pandemic, Public health laboratory services

## Abstract

**Background:**

Brazil has one of the highest numbers of COVID-19 cases and deaths. Rio Grande do Sul (RS) in southern Brazil is one of the leading states in terms of case numbers. As part of the national public health network, the State Central Laboratory (LACEN-RS) changed its routine in 2020 to focus on the diagnosis of COVID-19. This study evaluated the laboratory surveillance of COVID-19 suspected cases analyzed at the LACEN-RS in 2020.

**Methods:**

Viral detection was performed using RT-qPCR in samples from patients with respiratory infection who met the study criteria. Viral RNA was isolated using commercial manual kits or automated extractors, and SARS-CoV-2 RT-qPCR was performed using the Bio-Manguinhos/Rio de Janeiro, IBMP/Paraná, or Allplex 2019-nCoV assay. In total, 360 representative SARS-CoV-2 samples were sequenced using the Illumina platform.

**Results:**

In total, 31,197 of 107,578 (positivity rate = 29%) tested positive for SARS-CoV-2. The number of RT-qPCR tests performed per month followed the COVID-19 epidemic curve observed for the state, with peaks in July-August and December. Females accounted for 63% of the samples, whereas the positivity rate was higher among males (33.1% males vs. 26.5% females). The positivity rate was higher in adults aged 50-79 years compared to the overall positivity rate. The majority of cases were observed in the capital, Porto Alegre, and the metropolitan region. Ten distinct lineages were identified, with B.1.1.28, B.1.1.33, and P.2 being the most frequent.

**Conclusions:**

Here, we describe laboratory surveillance of COVID-19 to identify priorities for epidemiological surveillance actions in RS.

## INTRODUCTION

Since December 2019, SARS-CoV-2 has infected millions of people worldwide, causing COVID-19^1^ ^-^ ^3^. According to international recommendations for diagnosis of SARS-CoV-2 infection, RT-qPCR analysis is the gold standard for detecting the virus in respiratory secretions^4^. 

Brazil has the third largest number of COVID-19 confirmed cases, followed by the number of deaths^5^. Rio Grande do Sul (RS), the southernmost Brazilian state, has a high annual incidence of respiratory viral infections^6^ ^,^ ^7^. Accordingly, the number of COVID-19 cases notified in RS in 2020 was high, totaling 430,780 reported cases, representing an overall incidence of 3,786.3 cases/100,000 inhabitants, despite all government efforts to control the pandemic. 

The Laboratory of Respiratory Viruses of the State Central Laboratory (LACEN-RS) is part of the National Public Health Network for the laboratory diagnosis of Influenza A, Influenza B, Parainfluenza virus 1-3, Adenovirus, and Respiratory Syncytial Virus^6^ ^,^ ^8^ ^-^ ^10^. In addition, some samples were sent to the National Reference Laboratory, Fundação Oswaldo Cruz (FIOCRUZ-RJ) for viral culture, immunological tests, and genotyping as part of the global surveillance of respiratory viruses^6^ ^,^ ^11^. With the global increase in the number of COVID-19 cases, the Brazilian Ministry of Health declared COVID-19 a Public Health Emergency of National Importance in February 2020^12^. It is noteworthy that until April 30, 2020, cases of respiratory infection were investigated at LACEN-RS for the presence of various respiratory viruses^13^; however, the COVID-19 pandemic overburdened health services. Therefore, diagnosis of COVID-19 was prioritized, and LACEN-RS had to change its routine and focus on investigation of SARS-CoV-2 in samples received during the pandemic. 

To overcome the drastic increase in COVID-19 cases in the RS, the State Health Secretariat, with the help of LACEN-RS, a decentralized part of the laboratory diagnosis of SARS-COV-2; with that, other public and private institutions also became involved in the diagnosis for case confirmation. In addition, the Brazilian Ministry of Health implemented public platforms in other Brazilian states to expand testing for asymptomatic or mild cases (Testar/RS)^14^ ^,^ ^15^. 

LACEN/CEVS/SES-RS plays an essential role as a public entity specialized in laboratory surveillance and with expertise in respiratory viruses, whose routine could generate important data with key variables to assess and characterize the ongoing pandemic, such as sex, age, number of samples processed per month, positivity rate, municipality, and state region most affected, among others. Thus, our study evaluated the characteristics of COVID-19 suspected cases that were analyzed for investigation of SARS-CoV-2 by RT-qPCR during the first year of the pandemic. 

## METHODS

**Study design and data sources.** We conducted a retrospective cohort review of all suspected cases of SARS-CoV-2 infection, whose clinical samples were analyzed at LACEN-RS in 2020 (the beginning of the COVID-19 pandemic). We also assessed surveillance data related to COVID-19 reported to the Epidemiological Surveillance Office of (Centro Estadual de Vigilância em Saúde - CEVS). Laboratory data were accessed using the Laboratory Environment Manager (GAL) system, as recommended by the Brazilian Ministry of Health^6^. It can be accessed by health units, hospitals, epidemiological surveillance institutions, and the Ministry of Health. For comparison and analysis purposes, data related to the total number of cases in the RS, notified by other institutions, were also assessed^16^. Notification was based on the clinical and laboratory case definitions used by the Brazilian Ministry of Health^17^. Demographic and clinical characteristics of patients were collected at healthcare units and hospitals and then reported to the e-SUS Notifica System and the Sivep-Gripe System (Influenza Epidemiological Surveillance Information System) at the regional level. The variables analyzed were month of testing, sample type, gender, age, death outcome, municipalities, Regional Health Coordination (RHC), Health Macro-regions, RT-qPCR results, and SARS-CoV-2 lineage.

**Clinical samples.** Reactive testing for SARS-CoV-2 detection was performed in samples from patients with respiratory infection collected in RS who met at least one of the following criteria: symptoms of respiratory infection; hospitalization; outbreak investigations; death cases; health or security professionals; potential organ donors or transplant patients; patients or workers of Long-Term Care Institutions; indigenous; pregnant; and individuals in close contact with a SARS-CoV-2 positive individual. Clinical samples, including nasopharyngeal swabs (NPS), oropharyngeal swabs, nasopharyngeal aspirates, bronchoalveolar lavage fluid, and saliva, were collected from basic health units at public or affiliated hospitals in all 497 RS municipalities and sent to LACEN-RS for processing. Rules for collecting, storing, and transporting clinical samples have been implemented in accordance with the recommendations of the Ministry of Health since the beginning of Influenza surveillance^6^. Laboratory test results were reported by the LACEN-RS using the GAL system. 

**RNA isolation and purification.** Viral RNA was isolated from the samples using the PureLink Viral RNA/DNA Mini Kit (Invitrogen, Life Technologies, Carlsbad, CA), QIAamp Viral RNA Mini Kit (Qiagen, Hilden, Germany), or BIO GENE Viral DNA/RNA Extraction (Bioclin-Quibasa, Belo Horizonte, Brazil). After the acquisition of automated extractors, extractions were performed using either the King Fisher Flex System (Thermo Fisher Scientific) or Extracta 96 (Loccus). All protocols were performed in accordance with the manufacturer’s instructions. 

**Virus detection.** SARS-CoV-2 detection was based on RT-qPCR using either the Bio-Manguinhos SARS-CoV-2 (Rio de Janeiro, Brazil), IBMP (Curitiba, Brazil), or Allplex 2019-nCoV Assay (Seegene, Seoul, South Korea), according to the manufacturer’s instructions. Amplification was performed in either a 7500 Real-Time PCR System (Applied Biosystems-Life Technologies, Carlsbad, CA, USA) or a CFX Opus 96 Real-Time PCR System (Bio-Rad, Hercules, CA). 

**Good laboratory practices and quality control.** LACEN-RS is a reference laboratory, and all analyses follow good laboratory practices, quality control, and manufacturers' recommendations at all process stages to minimize doubtful results.

**Positivity Rate Analysis.** The positivity rate (PR) was calculated as the total number of all investigated cases that were positive for SARS-CoV-2 in relation to the total number of investigated cases (positive tests/total tests) × 100%^18^ ^-^ ^20^. The PR was calculated considering all samples analyzed at LACEN-RS as well as samples investigated elsewhere in RS, for each month and for the following variables: sample type, sex, age, outcome, municipality, RHC, and Health Macro-regions. 

**SARS-CoV-2 lineage circulation analysis.** 360 positive SARS-CoV-2 samples representative from all 18 RHC with Ct values threshold of ≤28^21^ for each epidemiological data were avaible^16^ were selected considering at least one of the following criteria: hospitalized patients, patients with death outcome, or index cases of each region. Genomic sequencing was performed at the Reference Laboratories Fundação Oswaldo Cruz (FIOCRUZ-RJ) and Fundação Ezequiel Dias (FUNED-MG) using Illumina sequencing protocols as previously described^22^ ^,^ ^23^; bioinformatic analysis was conducted using CLC Genomics Workbench version 20.0.4 (Qiagen A/S, Denmark). The metadata were sent back to the LACEN-RS with the SARS-CoV-2 lineage according to the COVID-19 Genomic Surveillance Regional Network^19^. All the genomes obtained in this study were uploaded to the EpiCoV database in the GISAID initiative^24^.

**Statistical analysis.** MedCalc ® v20.009 for Windows was used for statistical analyses. The chi-square test was used to compare two proportions (from independent samples), expressed as percentages with their respective 95% confidence intervals (95%CI). The significance level was set at p < 0.05.

**Ethics.** This study was approved by the Ethics Committee of the UFCSPA (protocol no. 3.978.647, CAAE 30714520.0.0000.5345).

## RESULTS

In this study, 108, 140 samples received at the LACEN-RS were analyzed for SARS-CoV-2 diagnosis in 2020. Of these, 562 samples presented problems related to collection, packaging, or identification and were excluded from the analysis, totaling 107,578 samples, of which 31,197 (29.0%) tested positive for SARS-CoV-2.

**FIGURE 1:**
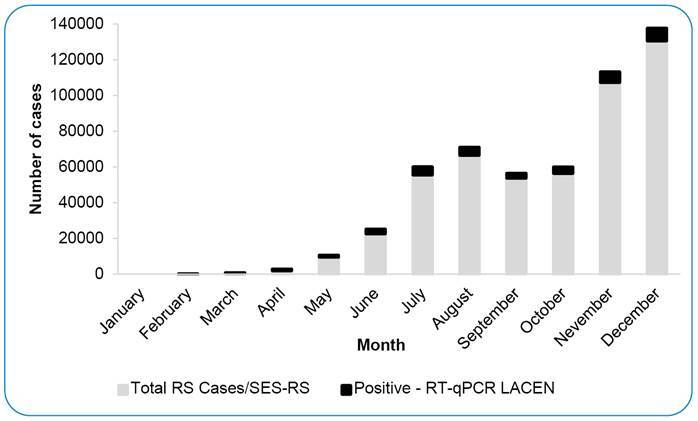
COVID-19 cases occurred in RS in 2020. The monthly SARS-CoV-2 positive cases analyzed at LACEN-RS (black) and monthly confirmed cases of SARS-CoV-2 infection in RS, according to the Ministry of Health criteria (grey).

The number of RT-qPCR tests conducted per month at LACEN-RS in 2020 increased significantly, from 160 in January to 18,650 in December, with the first SARS-CoV-2 positive case detected on February^25^. This increase followed the COVID-19 epidemic curve observed for the state, with peaks in July-August and December (Figure 1).

During the first pandemic months, from March to late April ***,*** all suspected cases of SARS-CoV-2 infection were diagnosed at LACEN-RS. As new laboratories became involved and other diagnostic methods (e.g., rapid COVID-19 antigen test) were incorporated into the state surveillance network, the percentage of samples sent to LACEN-RS decreased throughout the year, reaching 6.8% on average, ranging from 4.6% to 10.4%. As of September 2020, the laboratory has performed approximately 15,000 tests per month, with the highest amount in December 2020 (n=20,000). 

**FIGURE 2:**
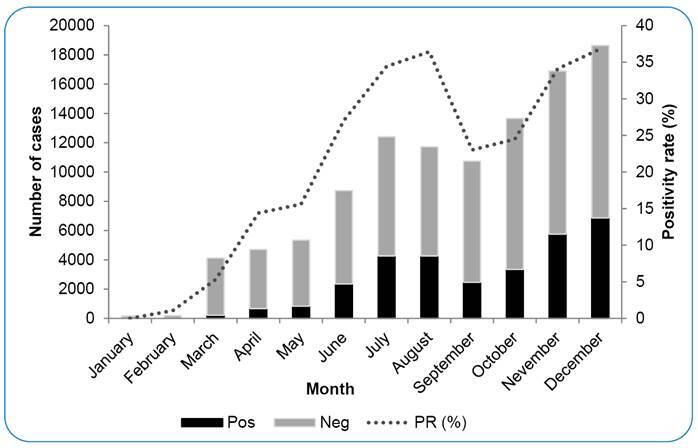
RT-qPCR tests performed in 2020 at LACEN-RS for diagnosis of SARS-CoV-2 infection and the positivity rate (PR).

A total of 2,379,592 suspected COVID-19 cases were investigated in RS in 2020, of which 518,603 (20.8%) were confirmed to be SARS-CoV-2 positive. Accordingly, the overall RS positivity rate was lower than the positivity rate obtained for samples analyzed at LACEN-RS (20.8% vs. 29.0%; 95% CI, 7.92 to 8.48) (Table 1). Of note, August and December had the highest positivity (36.4% and 36.8%, respectively) (Figure 2 and Table 1). NPS accounted for 75.8% of all samples analyzed at LACEN-RS; the positivity rate was 31.5% for NPS and 21.5% for the other samples (95% CI, 9.81 to 10.99) (Table 1). 

**TABLE 1:** Positivity rate (PR) of samples tested at LACEN-RS in 2020 for diagnosis of SARS-CoV-2 infection.

	TOTAL	VARIA-BLE	SARS-CoV-2 RT-qPCR or investigated COVID-19			TOTAL	%	PR (%)	Rate (95%CI)
			POS	NEG	D				
RS investi-gated cases	2,433,048		518,603	1,914,445	-	2,433,048		20.8	8.2 (7.92, 8.48)
LACEN RT-qPCR	107,578		31,197	76,381	562	107,578		**29.0**	
Monthly testing routine	107,354	Jan.	0	160		160		0	29.0 (26.64, 29.27)
		Feb.	2	186		188		1.1	27.9 (25.14, 28.74)
		Mar.	219	3,907		4,126		5.3	23.7 (22.92, 24.40)
		Apr.	678	4,031		4,709		14.4	14.6 (13.53, 15.61)
		May	834	4,514		5,348		15.6	13.4 (12.37, 14.39)
		Jun.	2,361	6,373		8,734		27.0	2.0 (1.02, 2.96)
		Jul.	4,272	8,135		12,407		34.4	5.4 (4.52, 6.28)
		**Aug.**	4,271	7,451		11,722		**36.4**	7.4 (6.49, 8,32)
		Sep.	2,473	8,272		10,745		23.0	6.0 (5.14, 6.8)
		Oct.	3,344	10,317		13,661		24.5	4.5 (3.72, 5.26)
		Nov.	5,764	11,140		16,904		34.1	5.1 (4.34, 5.87)
		**Dec.**	6,868	11,782		18,650		**36.8**	7.8 (7.06, 8.55)
Biological Samples	107,612	**NPS**	25,670	55,896	4	81,566	75.80	**31.5**	10.4 (9.81, 10.99)
		Others	5,485	20,253	304	25,738	24.20	21.5	
Gender	107,612	Female	18,036	49,788	11,541	67,824	63.02	26.5	4.6 (4.04, 5.16)
		**Male**	13,107	26,377	8,747	39,484	36.69	**33.1**	
Deaths	974	Female	117	331		448	46.00	26.12	0.5 (-5.07, 6.01)
		Male	140	386	1	526	54.00	26.62	

**PR:** positivity rate; **CI:** Confidence Interval; LACEN-RS positivity rate vs. variable positivity rate.

**FIGURE 3:**
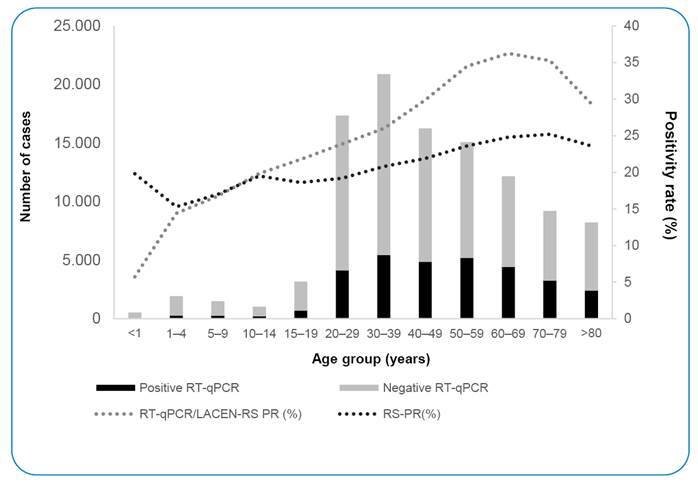
RT-qPCR tests performed according to age group and positivity rate (PR) for LACEN-RS and RS notified cases.

Females accounted for 63.0%, whereas the positivity rate was higher among males (33.1% males vs. 26.5% females; 95% CI, 4.04 to 5.16) (Table 1). The median age of the patients was 44 years (range, 0-100 years). The positivity rate was higher in adults aged 50-79 years compared to the overall positivity (50-59y:95% CI, 4.70 to 6.31; 60; 60-69y:95% CI, 6.31 to 8.10; 70-79y:95% CI, 5.29 to 7.32), whereas the lowest positivity rate was observed in children younger than 1 year (Figure 3 and Supplementary Table 1). 

Next, we analyzed the positivity rate for each age group in August and December 2020, which were the months with the highest positivity rates. Interestingly, in children and adolescents, the positivity rate was higher in August than in December, whereas in adults older than 40 years, the positivity rate was higher in December; in adults aged 15-39 years, no significant changes were observed in the positivity rate (Supplementary Table 1). 

Among all samples received at LACEN-RS, 0.9% (974/107,578) were declared deceased in the records. Among them, 26.2% (255/974) were positive for SARS-CoV-2. Females accounted for 46.0% (448/974) of death cases, with a positivity rate of 26.1% vs. 26.6% (526/974, 54.0%) in males. Moreover, most deceased patients were older than 50 years (85.5% of all females and 87.3% of all males in this group), and the positivity rate in this age group was 36.1% for females and 39.0% for males (Table 1 and Supplementary Table 2).

**FIGURE 4:**
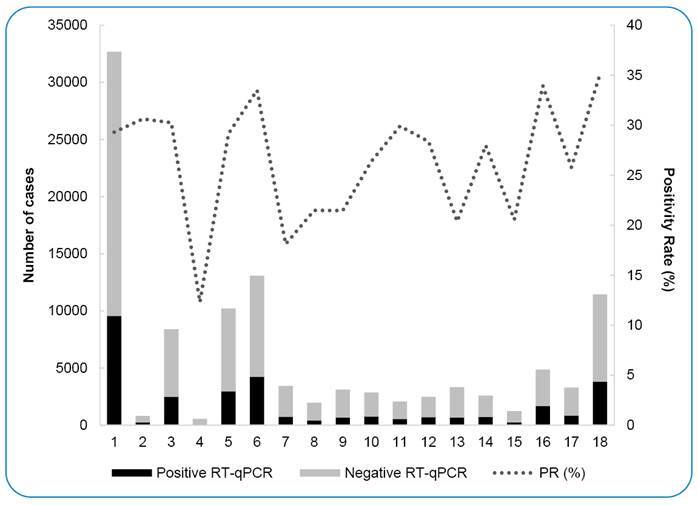
RT-qPCR tests performed according to 18 Regional Health Coordinators.

Samples analyzed at LACEN-RS were from 497 municipalities in RS, representing all 18 Regional Health Coordination's (RHC) of the state. RHC1, which includes the metropolitan region of Porto Alegre, accounted for the largest number of samples analyzed (n = 32,825), followed by RHC6 (Passo Fundo region), and RHC18 (Coast region), with 13,096 and 11,493 samples, respectively. The RHC with the lowest number of cases analyzed at LACEN-RS was RHC4 (Santa Maria region, n = 556). With regard to positivity, RHC18 presented the highest percentage of positive cases (35.1%), followed by RHC16 (Lajeado region), and RHC6 (33.9% and 33.5 %, respectively) (Figure 4 and Supplementary Table 3). 

Most samples analyzed at LACEN-RS were from residents of the state capital Porto Alegre (n=12,090; positivity rate of 26.3%), followed by residents of Passo Fundo (n=6,774; positivity rate 33.8%) and Pelotas (n=6,006; positivity rate 33.3%). Accordingly, considering the seven Health Macro-regions of RS (M/E, Metropolitan/East; CW, Center-West; M/NW, Missionary/Northwest; M, Mountains; N, North; S, South; V, Valley), M/E accounted for the highest number of residents whose samples were analyzed at LACEN-RS (n=43,399), whereas CW was the region with the lowest (n=3,405). The highest percentage of positive results was detected in the N region (30.9%), followed by M/E (30.4%) (Supplementary Table 3). 

In addition to samples collected from residents of RS, 420 samples from individuals who were visiting RS but residing in 24 other Brazilian states and three from other countries were also investigated at LACEN-RS, 27.9% of which were positive for SARS-CoV-2.

In total, 360 SARS-CoV-2 genome sequences were available in the GISAID database, and 10 distinct lineages were identified (Supplementary Table 4). Samples were selected based on epidemiological criteria to verify the current lineages. Considering the cumulative number of cases with lineage determination, until August 2020 B.1.1.33 was the predominant lineage (53.1%) in RS, followed by B.1.1.28 (22.3%). As of December 2020, with the introduction of the P.2 lineage in RS (25% of the samples analyzed), the representativeness of B.1.1.33 decreased to 31.1%, whereas that of lineage B.1.1.28 increased (30%).

## DISCUSSION

The analysis of SARS-CoV-2 surveillance at LACEN-RS allows a detailed assessment of the laboratory’s routine, helps improve service provision, and contributes to the control of respiratory viruses in RS. At the beginning of 2020, LACEN-RS had only five employees and two interns involved in the analysis of the respiratory viruses. To overcome such limited human resources to handle all pandemic demands, which was just beginning at that time, LACEN-RS established the SARS-CoV-2 RT-qPCR taskforce on March 6, 2020. 

The testing strategy for the diagnosis of SARS-CoV-2 in RS was formulated and implemented by the State Health Department following national and international guidelines and evolved logistically as the pandemic progressed throughout the country. New employees were recruited from several specialists who applied for positions through public open calls. Currently, LACEN-RS has five new employees, two temporary hires from the Pan American Health Organization (PAHO), seven employees from other institutions who are temporarily working at LACEN-RS, and 10 interns. In addition, logistics were reorganized, with improvements in the Receipt of Biological Samples unit, and partnership with the Center for Scientific and Technological Development of the State Center for Epidemiological Surveillance (CDCT/CEVS) for RT-qPCR testing, and with the State Committee for Epidemic Control (COERS/CEVS). In addition to investments in human resources, there were also investments in laboratory infrastructure to overcome the daily routine of examinations. Accordingly, LACEN-RS acquired commercial RT-qPCR kits on a large scale, thermocyclers, and automated nucleic acid extractors with funds from the State Secretariat of Health and Brazilian Ministry of Health. 

In 2020 and 2021, the number of available tests increased over time, and investment and technical improvements resulted in approximately 85.0% of LACEN-RS diagnosis results being released within 24 hours, and the remaining within 48 hours from sample reception. Timeliness diagnosis is paramount for pandemic control. Moreover, monitoring the positivity rate is one of the most important epidemiological tools for monitoring trends and disease magnitude for surveillance purposes and public health decision-making^19^ ^,^ ^20^ ^,^ ^26^. Notably, in places where positive rates are high, the number of confirmed cases is likely to represent only a small fraction of the true number of infections. Therefore, the epidemic is only considered under control when the positive rate reaches values below 5.0% for a minimum period of 14 days^18^.

In this study, the overall positivity rate was 29.0% (31,197/107,578), reaching 36.8% in August (first peak), and 36.4% in December (second peak). This increase in positive COVID-19 cases in RS coincided temporally with the epidemic situation in Brazil. However, in RS, the increase in the number of cases in the first peak occurred at least 15 days later than that in the rest of the country. In Brazil, the first peak was around mid-July and mid-August (epidemiological week 29-33), and the second peak was between late November and December (epidemiological week 49-52)^27^.

Our data show that after March 2020, when the positivity rate in RS was 5.3%, the COVID-19 epidemic scenario in RS only got worse^16^, despite all measures to control viral transmission recommendations, such as use of masks, social distancing, and hand hygienization^28^ ^-^ ^30^. Moreover, very high-risk transmission has been observed in the state since April 2020 (positivity rate 14.4%)^26^. This situation has been reinforced by data from FIOCRUZ-RJ for RS and other Federation Units (FUs), such as Bahia, Rio de Janeiro, São Paulo, and Mato Grosso do Sul. In this sense, trends of maintenance of positivity at relatively high values (25.0-50.0%) during the last weeks of December 2020 were observed in these states^20^. Another study in Brazil showed an overall RT-qPCR positivity rate of 44.6% in Rio de Janeiro^31^. 

On August 24, 2021, RS performed an average of 17.2 RT-qPCR tests per 100,000 people (1,978,493 tests for an 11,466,630 estimated population)^16^ ^,^ ^32^. São Paulo had 20.5 tests per 100,000 people (9,560,925 for 46,649,132 estimated population)^32^ ^,^ ^33^, while in another survey, a low coverage of RT-qPCR tests was observed in states such as Rio de Janeiro, Paraíba, Maranhão, Minas Gerais, and Pará^20^. 

LACEN-RS receives biological samples from different health center units/hospitals, and most are NPS (75.8%) because of the ease of collection and minimal-risk aerosol exposure to healthcare workers compared with invasive procedures to obtain other samples such as bronchoalveolar fluid^34^ ^,^ ^35^. A higher positivity rate of SARS-CoV-2 detection was observed in NPS (31.5%) than in the other samples (21.5%). Other studies suggest that NPS should be preferred over other specimens for detecting SARS-CoV-2, explaining that the higher positivity rate of NPS could be correlated to a higher viral load in the nasopharynx than other anatomical sites or specimens^35^ ^-^ ^40^. 

Regarding sex, we observed a higher number of samples from females than from males analyzed in the laboratory. In contrast, the positivity rate was significantly higher for males. This finding was different from the data of COVID-19 cases from the state database, which showed that more women were infected than men (63.0% vs. 47.0%). On the other hand, our data are in accordance with the national trend, with more cases reported among males compared among females^24^. In agreement with our results, a study in Rio de Janeiro found SARS-CoV-2 positivity to be higher among males, with a positivity rate of 52.6% in the metropolitan region^25^. In general, the labor market is mostly composed of men; therefore, men are usually more exposed to infection. 

Most samples analyzed at LACEN-RS were obtained from hospitalized patients and healthcare professionals. Regarding age, most individuals were 30-39 years; however, the highest positivity rate was observed among those aged 60-69 years. It is noteworthy that RS is the Brazilian state with the highest percentage of individuals older than 60 years (18.2%). Before the availability of vaccines for COVID-19, the elderly represented a large proportion of the individuals hospitalized with symptoms of respiratory infection in the state, with 85.6% hospitalization and 26.8% of deaths^41^, which explains the high positivity rate observed in this age group^42^ ^-^ ^45^. However, individuals in the age group of 20-49 years are professionally active and as a result, susceptible to infection. Finally, the positivity rate differences between age groups found in this study followed a pattern similar to that observed for the state^16^.

Samples from patients who died accounted for 0.9% of all the samples tested at the LACEN-RS in 2020. According to the Centers for Disease Control and Prevention (CDC), RT-qPCR remains the gold standard for clinical diagnostic detection of SARS-CoV-2 in postmortem specimens from deceased individuals with confirmed or suspected COVID-19, and studies have shown that SARS-CoV-2 RNA can be detected in pharynx samples up to 128 h after death, with only a small decrease in positivity and that SARS-CoV-2 viral load varies depending on the course of disease^46^ ^,^ ^47^. Accordingly, in our study, the positivity rate for samples from deceased patients was similar to that of the overall positivity rate (26.2% and 29.0%, respectively).

Most samples analyzed in our study were from RHC1, where Porto Alegre, the state capital, is located. With an estimated population of 2,369,210 inhabitants (20.8% of the RS population), Porto Alegre manages a health system for approximately 1,488,252 people, including residents of other municipalities^48^. 

RHCs 3, 6, and 16 also accounted for a large number of samples analyzed at the LACEN-RS. Many samples were from outbreaks in the meatpacking industries of the municipalities of Lajeado (RHC16) and Passo Fundo (RHC6). These meatpacking industries did not interrupt their production activities during the pandemic and were the target of numerous outbreaks throughout 2020^41^. When the state was ranked as medium risk for COVID-19, the Department of Health reported a series of 30 outbreaks in local industries. Of approximately 30,000 workers, approximately 3,000 had flu-like symptoms, and 611 (21.97%) tested positive for SARS-CoV-2. In addition, Lajeado and Passo Fundo are references for healthcare services in the region; hence, many samples from hospitals were also sent to the LACEN-RS by these municipalities. These RHCs also reported 11 outbreaks in closed institutions (homes for the elderly, prisons, and orphanages), with 475 symptomatic individuals investigated, confirming 141 (29.6%) cases^41^. Finally, RHC3, in the southern region of the state, includes the municipality of Pelotas, which is the fourth most populous city in RS, with several universities and reference hospitals that attend residents from the surroundings^16^.

The coastal region showed an increase in the number of positive cases after the flexibilization of the state-controlled distance system at the beginning of August 2020. In addition, with the arrival of spring in October and during summer, social distancing decreased as people traveled to other cities for vacations. As a result, the number of RT-qPCR positive cases increased from approximately 200 in August to almost 1,000 per month during spring-summer, when the state was ranked as high risk for COVID-19 transmission^41^.

The samples submitted by LACEN-RS for genetic characterization were representative of the SARS-CoV-2 scenario in RS, as the proportion of lineages was similar to that of all RS samples submitted to GISAID (**Supplementary Table 3**). As observed in RS and the rest of the country, with the emergence of B.1.1.28, B.1.1.33, and P.2, the number of cases increased due to the high transmissibility of these variants^49^ ^-^ ^53^. Using this collection, we found no association between the most frequent variants and any specific demographic data. The chosen biological samples were very stratified and difficult to relate to each other, since sequencing was performed only for samples that met the officially recommended criteria, as described previously. The analysis of SARS-CoV-2 lineages circulating during the pandemic period emphasizes the importance of genomic surveillance along with epidemiological data for a better understanding of the dynamics of virus transmission, which is paramount for guiding public health service decisions. 

This study has some limitations regarding low testing in the state and laboratory capacity, which obliged us to follow strict criteria for testing samples received at the LACEN-RS. Due to the scarce resources of the state and the country and global difficulties in the acquisition of lab supplies, LACEN-RS worked as a strategic spot for disease confirmation (severe cases, deaths, outbreaks, health professionals, etc.). Although testing in the state in 2020 was less than ideal and varied over the months, the representativeness of the samples received by LACEN-RS reflected the disease scenario in our state. 

In 2020, LACEN-RS helped in the implementation of SARS-CoV-2 diagnosis in 30 partner institutions (public and private), in the form of training, flow guidelines, and distribution of extraction/amplification kits in collaboration with the Ministry of Health. Fortunately, a large task force was set up in RS, bringing together public and private universities, research institutes, and public and private laboratories with the exchange of information and inputs to meet the SARS-CoV-2 pandemic. Undoubtedly, this remains the legacy of the COVID-19 pandemic.

Finally, this report is the first and largest study of SARS-CoV-2 RT-qPCR detection by sample, age, sex, time, and region in Rio Grande do Sul state during the first year of the COVID-19 pandemic. The laboratory surveillance results using positivity rates by geographic area help to identify priorities for control measures such as lockdown, social distancing, and vaccination.

## SUPPLEMENTARY MATERIAL

**SUPPLEMENTARY TABLE 1:** Comparison of age groups of SARS-CoV-2 cases investigated at LACEN-RS and RS during 2020.

					LACEN SAMPLES TESTED				RS INVESTIGATED CASES			
Age (y)	Pos	Neg	Total	R	PR (%)	Rate (95%CI)	Pos	Neg	Total	R	PR (%)	Rate (95%CI)
**Under 1**	30	493	523	12	5.7	23.30 (20.95, 25.01)	1,924	7,788	9,712	12	19.8	1.50 (0.69, 2.28)
**1-4**	278	1,652	1,930	9	14.4	14.60 (12.94, 16.12)	6,857	37,916	44,773	9	15.3	6.00 (5.66, 6.33)
**5-9**	251	1,246	1,497	10	16.8	12.20 (10.20, 14.03)	7,896	38,449	46,345	11	17.0	4.30 (3.95, 4.64)
**10-14**	205	828	1,033	11	19.8	9.20 (6.64, 11.53)	9,255	38,107	47,362	10	19.5	1.80 (1.44, 2.16)
**15-19**	689	2,476	3,165	8	21.8	7.20 (5.70, 8.63)	19,490	85,021	104,511	6	18.6	2.70 (2.46, 2.94)
**20-29**	4,140	13,199	17,339	2	23.9	5.10 (4.40, 5.78)	96,843	407,543	504,386	2	19.2	2.10 (1.98, 2.22)
30-39	5,425	15,460	20,885	1	26.0	3.00 (2.34, 3.65)	117,425	446,494	563,919	1	20.8	0.50 (0.38, 0.62)
**40-49**	4,857	11,389	16,246	3	29.9	0.90 (0.15, 1.65)	95,789	342,097	437,886	3	21.9	0.60 (0.47, 0.73)
**50-59**	5,202	9,883	15,085	4	34.5	5.5 0(4.70, 6.31)	77,001	249,250	326,251	4	23.6	2.30 (2.14, 2.45)
**60-69**	4,408	7,755	12,163	5	**36.2**	7.20 (6.31, 8.10)	49,300	149,142	198,442	5	24.8	3.50 (3.30, 3.70)
70-79	3,244	5,956	9,200	6	35.3	6.30 (5.29, 7.32)	24,191	71,814	96,005	7	**25.2**	3.90 (3.62, 4.18)
**≥ 80**	2,412	5,802	8,214	7	29.4	0.40 (-0.61, 1.43)	12,632	40,824	53,456	8	23.6	2.30 (1.94, 2.66)
**Total**	31,141	76,139	107,280	-	**29.0**	-	518,603	1,914,445	2,433,048	-	**21.3**	7.70 (7.42, 7.98)

PR: positivity rate; R: ranking from highest to lowest number of samples/cases tested/investigated; y: years; CI: Confidence Interval.

## Appendix

**SUPPLEMENTARY TABLE 2:** Samples declared in laboratory registration documents as deceased patients analyzed at LACEN-RS in 2020, distributed by age group and sex.

Age (y)					Female				Male			
	Pos	Neg	Total	PR (%)	Pos	Neg	Total	PR (%)	Pos	Neg	Total	PR (%)
**Under 1**	0	5	5	0.0	0	0	0	0.0	0	5	5	0.0
**1-4**	1	5	6	16.7	1	3	4	25.0	2	0	2	100.0
**5-9**	0	1	1	0.0	0	0	0	0.0	0	1	1	0.0
**10-14**	0	2	2	0.0	0	2	2	0.0	0	0	0	0.0
**15-19**	2	3	5	40.0	0	2	2	0.0	2	1	3	66.7
**20-29**	1	11	12	8.3	1	10	11	9.1	0	1	1	0.0
**30-39**	4	38	42	9.5	3	17	20	15.0	1	21	22	4.5
**40-49**	11	48	59	18.6	5	21	26	19.2	6	27	33	18.2
**50-59**	30	88	118	25.4	14	28	42	33.3	16	60	76	21.0
**60-69**	59	140	199	29.6	26	46	72	**36.1**	33	94	127	25.9
**70-79**	65	195	260	25.0	24	80	104	23.1	41	115	156	26.3
**≥ 80**	82	183	265	30.9	43	122	165	26.1	39	61	100	**39.0**
**Total**	255	719	974	**26.2**	117	331	448	**26.1**	140	386	526	**26.6**

**PR:** positivity rate; **y:** years.

## Appendix

**SUPPLEMENTARY TABLE 3:** Provenance of samples analyzed at LACEN-RS in 2020.

	TOTAL	VARIA-BLE	SARS-CoV-2 RT-qPCR or investigated COVID-19			Total	PR (%)	Rate (95%CI)
			POS	NEG	D			
**RHC**	107,667	1ª	9,562	23,115	148	32,82	29.3	0.30 (-0.26, 0.86)
		2ª	249	561	3	813	30.6	1.60 (-1.48, 4.86)
		3ª	2494	5,902	23	8,419	30.3	1.30 (0.28, 2.34)
		4ª	66	489	1	556	12.3	16.70 (13.64, 19.23)
		5ª	2,969	7,250	17	10,23	29.1	0.10 (-0.81, 1.03)
		6ª	4,250	8,826	20	13,09	33.5	4.50 (3.65, 5.36)
		7ª	736	2,698	10	3,444	18.1	10.90 (9.65, 12.08)
		8ª	422	1,540	2	1,964	21.5	7.50 (5.61, 9.28)
		9ª	671	2,451	3	3,125	21.5	7.50 (6.00, 8.93)
		10ª	757	2,104	8	2,869	26.4	2.60 (0.93, 4.20)
		11ª	536	1,530	2	2,068	29.9	0.90 (-1.19, 3.08)
		12ª	707	1,781	4	2,492	28.4	0.60 (-1.22, 2.36)
		13ª	681	2,651	10	3,342	20.4	8.60 (7.17, 9.96)
		14ª	718	1,865	7	2,590	28.0	1.00 (-0.79, 2.72)
		15ª	254	976	5	1,235	20.6	8.40 (6.04, 10.58)
		16ª	1,690	3,176	16	4,882	33.9	4.90 (3.57, 6.25)
		17ª	852	2,444	7	3,303	25.8	3.20 (1.67, 4.69)
		18ª	3,811	7,638	14	11,46	35.1	6.10 (5.17, 7.04)
**Munic.of residence**	107,401	POA	3,183	8,864	43	12,09	**26.3**	2.70 (1,86, 3,52%)
		PF	2,291	4,475	8	6,774	**33.8**	4.80 (3,65, 5,97)
		PEL	2,003	3,993	10	6,006	**33.3**	4.30 (3,09, 5,53%)
**Health Macro-regions**	107,401	**M/E**	13,215	30,032	152	43,39	**30.4**	4.40 (3.88, 4.92)
		CW	821	2,575	9	3,405	24.1	4.90 (3.41, 6.33)
		S	3,286	9,050	32	12,36	26.6	2.40 (1.57, 3.22)
		**N**	5,118	11,384	30	16,53	**30.9**	1.90 (1.15, 2.66)
		M/NW	2,936	8,529	21	11,48	25.6	3.40 (2.55, 4.23)
		V	2,800	7,445	28	10,27	27.3	1.70 (0.79, 2.59)
		M	2,889	7,014	17	9,920	29.1	2.40 (1.54, 3.24)

**CI:** Confidence Interval; LACEN-RS positivity rate (29.0%) vs. variable positivity rate; **RHC:** regional health coordinators; **Munic. of residence:** municipality of residence; **POA:** Porto Alegre; **PF:** Passo Fundo; **PEL:** Pelotas; **M/E:** metropolitan/east; **CW:** center-west; **M/NW:** missionary/northwest; **M:** mountains; **N:** north; **S:** south; **V:** valleys; **D:** discarded samples.

## Appendix

**SUPPLEMENTARY TABLE:** 4: SARS-CoV-2 genome sequences and lineages of samples analyzed at LACEN-RS (A) and of all SARS-CoV-2 strains from RS available in the GISAID database (B).

A. SARS-CoV-2 genome sequences and identified lineages from LACEN-RS											
Lineage	Month										Total
	March	April	May	June	July	August	September	October	November	December	
**A**	1	0	0	0	0	0	0	0	0	0	1
**B**	1	0	0	0	0	0	0	0	0	0	**1**
**B.1**	7	0	0	0	0	0	0	0	1	0	**8**
**B.1.1**	3	2	4	0	3	7	1	0	0	0	**20**
**B.1.1.28**	7	1	3	4	6	**19**	1	3	44	**20**	**108**
**B.1.1.33**	7	5	29	12	17	25	2	3	8	4	**112**
**B.1.1.332**	0	0	0	0	1	1	0	0	0	0	**2**
**B.1.91**	0	0	4	3	5	1	0	0	4	0	**17**
**N.1**	0	0	0	0	0	1	0	0	0	0	**1**
**P.2**	0	0	0	0	0	0	0	2	60	28	**90**
**Total**	**26**	8	**40**	**23**	**32**	**54**	**4**	**8**	**117**	**52**	**360**
											
**B. SARS-CoV-2 genome sequences and identified lineages available in GISAID from RS.**											
	Month										
**Lineage**	March	**April**	**May**	**June**	**July**	**August**	**September**	**October**	**November**	December	Total
A	2	0	0	0	0	0	0	0	0	0	2
B	1	0	0	0	0	0	0	0	0	0	**1**
B.1	10	0	1	0	1	2	4	0	2	0	**20**
B.1.1	3	2	6	0	3	8	1	1	0	0	**24**
B.1.1.161	2	2	2	2	1	2	0	1	0	1	**13**
B.1.1.28	9	1	5	20	19	38	20	31	79	**26**	**248**
B.1.1.33	11	22	50	49	45	58	33	22	18	**6**	**314**
B.1.1.332	0	0	0	0	1	1	0	1	0	0	**3**
B.1.1.70	0	0	0	0	0	0	0	0	1	0	**1**
B.1.195	0	0	0	2	1	0	0	0	0	0	**3**
B.1.212	0	0	0	1	0	0	0	0	0	0	**1**
B.1.91	0	0	4	8	8	3	0	0	6	0	**29**
N.1	0	0	0	0	0	1	0	1	0	0	**2**
P.1	0	0	0	0	0	0	0	0	2	0	**2**
P.2	0	0	0	0	0	1	0	10	74	36	**121**
**Total**	**38**	**27**	**68**	**82**	**79**	**114**	**58**	**67**	**182**	**69**	**784**
